# Telehealth Care for People With Serious Illnesses and Preferred Languages Other Than English

**DOI:** 10.1001/jamanetworkopen.2025.29880

**Published:** 2025-09-03

**Authors:** Yidi Wang, Rebecca L. Sudore, Carly Zapata, Roman Martinez, Jasmine Gonzalez, Vincent Le, Mara Quinn, Carrie Huang, Claudia Perez-Vaughan, Steven Z. Pantilat, Courtney R. Lyles, Christine S. Ritchie, Sarah Nouri

**Affiliations:** 1 School of Medicine, University of California, San Francisco; 2 Division of Geriatrics, Department of Medicine, University of California, San Francisco; 3 Division of Palliative Medicine, Department of Medicine, University of California, San Francisco; 4 San Francisco Free Clinic, San Francisco, California; 5 Division of Palliative Care, Department of Medicine, Alameda Health System, Oakland, California; 6 Center for Healthcare Policy and Research, Department of Public Health Sciences, University of California, Davis, Sacramento; 7 Division of Palliative Care and Geriatric Medicine, Center for Aging and Serious Illness, Department of Medicine, Massachusetts General Hospital and Harvard Medical School, Boston

## Abstract

**Importance:**

Telehealth use in outpatient care has increased. Patients who speak preferred languages other than English (PLOE) experience disparities in telehealth use and in care provided via telehealth for serious illnesses (eg, cancer and other illnesses with high mortality risk and negative effects on daily functioning or quality of life). However, little is known about the experiences of people with PLOE receiving serious illness care via telehealth.

**Objective:**

To identify facilitators and barriers to providing serious illness care via telehealth to people with PLOE.

**Design, Setting, and Participants:**

This qualitative study analyzed semistructured interviews with Cantonese- or Spanish-speaking patients or their caregivers receiving outpatient serious illness care via telehealth at 2 University of California, San Francisco palliative care clinics (one focused on cancer and one on noncancer serious illnesses). The study included participants aged 18 years or older who spoke Spanish or Cantonese (the 2 most prevalent non-English languages in San Francisco). Participants were recruited via telephone outreach conducted by language-concordant study staff. If patients could not communicate by telephone (eg, they had anarthria or advanced dementia), their caregivers were interviewed instead. Semistructured interviews were conducted between June and August 2023.

**Exposure:**

At least 1 outpatient telehealth visit in the last year.

**Main Outcomes and Measures:**

One-on-one semistructured interviews developed using the Capability, Opportunity, Motivation, and Behavior framework were conducted by telephone to capture patient and caregiver perceptions on facilitators and barriers to serious illness care via telehealth. Interviews were recorded, transcribed, translated, and analyzed using thematic analysis.

**Results:**

This study included 20 participants (mean [SD] age, 64.7 [15.4] years; 14 females [70%]). Three key themes were identified from the transcripts of 9 Cantonese-speaking participants (45%; 3 patients and 6 caregivers) and 11 Spanish-speaking participants (55%; 7 patients and 4 caregivers). First, health system processes determined visit type and impacted telehealth accessibility. Second, between-visit communication using digital health tools (eg, the electronic patient portal) was challenging due to limited availability in non-English languages and digital literacy requirements. Finally, patient-clinician language concordance and longitudinal relationships with certified interpreters facilitated high-quality serious illness communication during telehealth visits, although family interpreters were sometimes required to overcome barriers associated with video interpretation, because patients sometimes had concerns about interpreter privacy and accuracy.

**Conclusions and Relevance:**

In this qualitative study of 20 semistructured interviews, Cantonese- and Spanish-speaking participants experienced barriers to serious illness care via telehealth before, during, and between visits. These findings underscore the need for interventions to promote equitable telehealth care for people with serious illnesses and PLOE, such as training more language-concordant clinicians, incorporating interpreters into interdisciplinary teams, and ensuring access to patient portals in languages other than English.

## Key Points

**Question:**

Are there facilitators and barriers to providing serious illness care via telehealth for people with preferred languages other than English?

**Findings:**

In this qualitative study of interviews with 20 Cantonese- and Spanish-speaking patients with serious illnesses or their caregivers, participants encountered linguistic challenges with digital health tools (eg, the electronic patient portal) used for previsit and between-visit communication. During telehealth visits, they valued certified interpreters but preferred language-concordant clinicians for better communication and cultural connection.

**Meaning:**

These findings suggest that linguistic inequity in serious illness care must be addressed using tailored interventions to overcome barriers related to digital health tools and certified interpreter use.

## Teaser

This qualitative study analyzes interviews with Cantonese- and Spanish-speaking patients or their caregivers to identify facilitators and barriers to providing serious illness care via telehealth to people with preferred languages other than English.

## Introduction

Telehealth use in outpatient care has increased substantially.^1,2,3^ Telehealth can facilitate increased access to care, especially for people with a serious illness, defined as having a health condition with a high mortality risk and negative effects on one’s daily functioning or quality of life.^4^ However, the use and usefulness of telehealth is low among certain populations, including people with preferred languages other than English (PLOE).^5,6,7^ Moreover, studies have shown that people with PLOE experience lower-quality patient-clinician communication,^6,8,9^ including less frequent goals-of-care discussions.^10^ The intersection of these disparities in telehealth and patient-clinician communication among people with PLOE with a serious illness is highly concerning, given that effective serious illness communication is a cornerstone of providing goal-concordant care. Yet little is known about the experiences of people with PLOE who receive serious illness care via telehealth.

Foundational to high-quality communication for people with PLOE are language concordance and interpretation. Receiving care from a language-concordant clinician—someone fluent in a patient’s preferred language—is associated with improved health education, increased patient satisfaction,^11,12^ and decreased adverse health outcomes in the primary care setting.^13^ In the absence of language-concordant clinicians, federal regulations require the availability of certified interpreters who have received professional training.^14^ However, family or other bilingual health care team members are often used as ad hoc interpreters instead of certified interpreters, which has been associated with worse prognostic understanding and symptom management for patients during end of life.^15^ Notably, existing research on experiences with and outcomes related to interpreter use has focused on in-person and primary care.^16^

There is a critical gap in understanding the experiences, facilitators, and barriers of serious illness care via telehealth among people with PLOE, including language concordance and interpretation. To address these gaps, we conducted a qualitative study with Spanish- and Cantonese-speaking patients or their caregivers receiving care at 2 outpatient palliative care clinics within a large, academic health center.

## Methods

This qualitative study was approved by the University of California, San Francisco (UCSF) Institutional Review Board. Data for this study comprised transcripts of semistructured interviews conducted between June and August 2023 as part of a larger study of patient experiences with serious illness care provided via telehealth by 2 UCSF palliative care clinics. One UCSF clinic focuses on cancer, and the other focuses on noncancer serious illnesses. Informed consent was obtained orally from study participants. Additional details are presented in the eMethods in Supplement 1. The study followed the Consolidated Criteria for Reporting Qualitative Research (COREQ) reporting guideline.

### Study Setting and Participants

Participants were selected through consecutive sampling of patients via telephone outreach conducted by language-concordant study staff. Patients were included if they were aged 18 years or older, spoke Spanish or Cantonese (the 2 most prevalent non-English languages in San Francisco), and had participated in at least 1 outpatient palliative care telehealth visit in the prior year. If patients were unable to communicate by telephone (eg, if they had anarthria or advanced dementia), we interviewed their caregivers instead. This decision reflected our clinical practice, in which visits are led by caregivers when patients are unable to communicate directly. Telehealth use in outpatient palliative care at UCSF increased from 50% to 100% at the start of the COVID-19 pandemic and has remained high (93%) since 2020. Initial visits are scheduled through the centralized UCSF contact center, where patients should be offered a choice between video and in-person visits. Follow-up visits are defaulted to telehealth unless patients or clinicians request an in-person appointment. Telehealth visits captured during the study period were conducted using Zoom videoconferencing (Zoom Video Communications), with certified interpreters (either UCSF staff or on-demand interpreters from an external company) joining by video.

### Data Collection Procedure

We used the Capability, Opportunity, Motivation, and Behavior (COM-B) theory^17^ to develop our interview guide assessing language-related experiences with digital health use in outpatient palliative care. We pilot tested the interview guide with our Community Advisory Board. Interviews were conducted via telephone by study staff who were native speakers; they were recorded and transcribed for analysis. Interviews in Cantonese were professionally translated to English before analysis; interviews in Spanish were analyzed in Spanish due to the fluency of our authorship team. We manually abstracted sociodemographic and clinical characteristics from the electronic health record, including age, language (Cantonese or Spanish), self-reported race and ethnicity (Asian or Hispanic), sex (male or female), diagnosis (cancer or noncancer serious illness), and electronic patient portal (MyChart; Epic) use. Race and ethnicity data were systematically collected as part of our larger study, which included English-speaking participants representing diverse racial and ethnic backgrounds, given the disparities in telehealth use and outpatient palliative care utilization related to racism and discrimination.

### Qualitative Analysis

Interviews were analyzed using thematic analysis^18^ in Dedoose, version 9.0 (Sociocultural Research Consultants LLC). We group coded a subset of interviews inductively to create 2 codebooks for Spanish and Cantonese transcripts that blended the COM-B capability, opportunity, and motivation domains around core processes that emerged. We then double coded 5 (25%) of the interviews. Given the more than 94% overlap between the 2 languages, we combined the codebooks into one and double coded the remaining transcripts. We met frequently to discuss emerging themes and resolve disagreements. To assess potential differences, we examined themes within and across language groups and between patients and caregivers.

## Results

### Participant Characteristics

We interviewed 20 participants (14 females [70%] and 6 males [30%]), with a mean age of 64.7 (15.4) years (Table 1). The 9 Cantonese-speaking participants (45%) included 3 patients and 6 caregivers, and all (100%) identified as Asian; 7 (78%) had cancer and 2 (22%) had noncancer serious illness. The 11 Spanish-speaking participants (55%) included 7 patients and 4 caregivers, and all (100%) identified as Hispanic; 8 (73%) had cancer and 3 (27%) had noncancer serious illness. All caregivers were family members with the same language preference as the patients they cared for.

**Table 1.** Participant Characteristics^a^

Characteristic	Cantonese-speaking participants (n = 9)	Spanish-speaking participants (n = 11)
Sex		
Male	3 (33)	3 (27)
Female	6 (67)	8 (73)
Age, mean (SD), y	73.5 (14.6)	57.5 (12.4)
Race and ethnicity		
Asian	9 (100)	0
Hispanic	0	11 (100)
Diagnosis		
Cancer	7 (78)	8 (73)
Noncancer serious illness	2 (22)	3 (27)
Interview type		
Patient	3 (33)	7 (64)
Caregiver	6 (67)	4 (36)
Electronic patient portal use, No./total No. (%)		
By patient	1/3 (33)	6/7 (86)
By caregiver	6/6 (100)	4/4 (100)
Received care from language-concordant clinician	1 (11)	3 (27)

^a^ Unless indicated otherwise, values are presented as No. (%) of participants.

There were notable differences in age, with Cantonese-speaking participants being older than Spanish-speaking participants (mean [SD], 73.5 [14.6] vs 57.5 [12.4] years). In addition, reported electronic patient portal use was lower among Cantonese-speaking patients compared with Spanish-speaking patients (1 of 3 [33%] vs 6 of 7 [86%]).

### Thematic Analysis

We identified 3 overarching themes that cut across all COM-B domains. Themes spanned the process of using telehealth for serious illness care as follows: (1) health system processes determine visit type and telehealth access, (2) barriers to between-visit communication, and (3) facilitators and barriers during telehealth visits. The findings for each theme are presented hereinafter with COM-B domains in parentheses (complete participant quotes are provided in Table 2).

**Table 2.** Themes, Subthemes, COM-B Elements, and Illustrative Quotations

Theme, subtheme, and COM-B element	Quotation
**Theme 1: health system processes determine visit type and telehealth access**	
Lack of agency is present in choosing visit type (capability, opportunity)	“I do wonder whether at some point they would send somebody just to have some in-person contact. And I don’t know if that’s because at this point in her care, they don’t feel it’s necessary or that’s just not something that they do. It’s hard to tell whether it’s because we’re remote or whether it’s because it’s a policy, you know, that it’s not required.” (Cantonese-speaking caregiver)
	“Since it was already scheduled [as a video visit], we just followed up on it. They just set up the appointment and we said, ‘Okay. Well, that’s what we have to do.’ So, that’s why we said we have to go through with it. We’re not going to say no.” (Spanish-speaking caregiver)
Usability of telehealth platform is a barrier to engagement (capability)	“My father is elderly … I had to help him with everything. I needed to set up Zoom, video calls, and assist him in downloading these apps because he has no idea how to operate them.” (Cantonese-speaking caregiver)
	“Doctor sometimes just sent a message, like, asking me to do a video chat. I need to tell my daughter to come over and help me set up.” (Cantonese-speaking patient)
**Theme 2: barriers to between-visit communication**	
Limited digital literacy is a barrier to patient portal use (capability)	“For people who don’t understand English well or who are too old or have no computer or phone knowledge, they might find it difficult to log in [to the patient portal]. …When I first started using it, I found it very difficult to log in. Today I know how to use it. But I still don’t know how to respond to messages because my English is limited. Sometimes I can find my daughter to help me, but she is only 15 years old and sometimes she had trouble understanding me.” (Cantonese-speaking caregiver)
Patient portal is perceived as useful, but lack of translation options limits its use (capability, opportunity)	“UC sends a message to him for each appointment. He sees that it mentions a date and time … but he doesn’t comprehend it because it’s all in English. He receives notifications indicating it might be a video appointment [or] walk-in. Sometimes he can’t distinguish between them. …If [the patient portal] could offer multiple language options, it would be even better.” (Cantonese-speaking caregiver)
	“I do everything through [the patient portal]. For me, it’s something that helps a lot. I can’t say that I completely understand it because, well, I only use it for what I need, but I see that it has so many features. If I had to make a suggestion, it would be that [the portal] should have a translation option.” (Spanish-speaking patient)
	“She [caregiver] contacts them because I’m not very proficient in English, so she interacts with the doctor. …Right now, what I find inconvenient is that I’m not very proficient in English. So, it’s difficult for me to seek medical care on my own. …If you were to make a phone call to me and use Chinese to communicate with me, it’s easy, in that case.” (Cantonese-speaking patient)
**Theme 3: facilitators and barriers during telehealth visits**	
Language concordance is ideal (capability, motivation)	“They [language-concordant physicians] understand us better because they know what we are talking about. …If the doctor spoke Spanish, they would understand me better because I’m giving 100%.” (Spanish-speaking patient)
	“It makes a big difference for privacy and the fluency of communication. I asked yesterday for a doctor who spoke Spanish, like my family doctor. …Sometimes it’s hard to explain to the interpreter.” (Spanish-speaking patient)
	“If it’s in your own language, it’s more suitable for you. … However, when there’s no language match, having an interpreter is still acceptable because, after all, we are in the United States, and there are situations where interpretation is necessary.” (Cantonese-speaking caregiver)
	“The [language-concordant] doctor has sometimes checked my fears… He tells me, ‘Don’t be afraid of this and that.’ That breaks barriers. We speak the same language so there is more I understand. Because if there is more understanding, there will be more care.” (Spanish-speaking patient)
Certified interpreters are essential in the absence of language-concordant clinicians (opportunity)	“It was OK if there were interpreters… I am satisfied if the interpretation services are available.” (Cantonese-speaking patient)
Longitudinal relationships with interpreters facilitate communication and alleviate privacy concerns (motivation)	“Sometimes your own interpreters, we’ve met them every time. So we knew each other well… After the nurse [is] done asking questions, when we were waiting for the doctor, we might chat with each other. They would tell us they are UC employees. They are very nice.” (Cantonese-speaking caregiver)
	“For one’s privacy, having to tell [interpreters] all the symptoms… Sometimes I don’t know where those people who are listening to one’s conversations are from.” (Spanish-speaking patient)
Duration, accuracy, and inability to accommodate diverse accents or hearing abilities are barriers to interpretation and lead to a preference for family interpreters (capability, opportunity)	“One appointment, originally half an hour, became an hour, and it’s not possible to be very detailed. …We translate ourselves and communication with the doctor becomes easier. …Translation has its benefits, but if it can be avoided we’d rather not use translation because we find it really a waste of time, not very accurate. What I want to say isn’t necessarily what is actually said.” (Cantonese-speaking caregiver)
	“Some of those interpreters translate very well. …Of course, when you hear it yourself and you say it yourself, it’s expressed better.” (Cantonese-speaking patient)
	“I will say I don’t use the interpreter team much … because my dad doesn’t hear well and sometimes we have people from other countries … and there is an accent. I mean, it’s difficult for my dad, you understand?… It becomes much easier and faster for me to do it myself.” (Spanish-speaking caregiver)

Abbreviations: COM-B, Capability, Opportunity, Motivation, and Behavior; UC, University of California.

#### Theme 1: Health System Processes Determine Visit Type and Telehealth Access

Participants perceived telehealth as the default visit type and had unvoiced questions regarding alternative visit types (opportunity). Notably, this theme was absent among English-speaking participants in the same study cohort.

“I do wonder whether at some point they would … have some in-person contact. …I don’t know if that’s because … they don’t feel it’s necessary or that’s just not something that they do. It’s hard to tell whether it’s because we’re remote or whether it’s because it’s a policy that it’s [in-person visit] not required.” (Cantonese-speaking caregiver)

“Since it was already scheduled [as a video visit], we just followed up on it. They just set up the appointment and we said, ‘Okay. Well, that’s what we have to do.’ …We’re not going to say no.” (Spanish-speaking caregiver)

Participants described that the usability of the telehealth video platform affected how accessible the telehealth visits were, often necessitating help from caregivers (capability):

“I had to help [my father] with everything. I needed to set up Zoom, video calls, and assist him in downloading these apps because he has no idea how to operate them.” (Cantonese-speaking caregiver)

#### Theme 2: Barriers to Between-Visit Communication

Navigating digital health tools such as the electronic patient portal can be technically challenging, even for caregivers, due to limited availability in non-English languages and digital literacy requirements (capability):

“For people who don’t understand English well…, they might find it difficult to log in. …I still don’t know how to respond to messages because my English is limited. Sometimes I can find my daughter to help me, but she is only 15 years old and sometimes she had trouble understanding me.” (Cantonese-speaking caregiver)

Although participants found the patient portal useful for accessing health information, they noted that its limited availability in different languages and lack of automatic translation negatively affected its usability and led to preferences for in-between-visit communication via telephone (capability, opportunity).

“I do everything through [the patient portal]. …I can’t say that I completely understand it… If I had to make a suggestion, it would be that [the portal] should have a translation option.” (Spanish-speaking patient)

“Right now, what I find inconvenient is that I’m not very proficient in English. So, it’s difficult for me to seek medical care on my own. …If you were to make a phone call to me and use Chinese to communicate with me, it’s easy, in that case.” (Cantonese-speaking patient)

#### Theme 3: Facilitators and Barriers During Telehealth Visits

Participants reported a preference for language-concordant clinicians vs interpreters during video visits because of enhanced communication clarity, understanding, and shared cultural connections that helped address participant fears and doubts more effectively. Based on these perceived benefits, some patients noted that having language-concordant clinicians can lead to better clinical care (capability, motivation):

“They [language-concordant physicians] understand us better because they know what we are talking about. …If the doctor spoke Spanish, they would understand me better because I’m giving 100%.” (Spanish-speaking patient)

“[A language-concordant physician] tells me, ‘Don’t be afraid of this and that.’ That breaks barriers. We speak the same language so there is more I understand. Because if there is more understanding, there will be more care.” (Spanish-speaking patient)

In the absence of language-concordant clinicians, participants named access to certified interpreters as an essential component of their video visits (opportunity):

“If it’s in your own language, it’s more suitable for you. …However, when there’s no language match, having an interpreter is still acceptable because, after all, we are in the United States, and there are situations where interpretation is necessary.” (Cantonese-speaking caregiver)

Although participants were generally comfortable discussing difficult topics in the presence of certified interpreters, they noted greater comfort with staff interpreters rather than externally hired interpreters, who are more frequently used due to scheduling logistics. Participants attributed this difference in comfort both to the opportunity to develop longitudinal relationships over time with staff interpreters and noted concerns about privacy and trust with externally hired interpreters (motivation):

“Sometimes your own interpreters, we’ve met them every time. So we knew each other well… After the nurse [is] done asking questions, when we were waiting for the doctor, we might chat with each other. They would tell us they are UC [University of California] employees. They are very nice.” (Cantonese-speaking caregiver)

“For one’s privacy, having to tell [interpreters] all the symptoms… Sometimes I don’t know where those people who are listening to one’s conversations are from.” (Spanish-speaking patient)

Participants noted that family interpreters were sometimes required to overcome barriers associated with video interpretation. Some participants noted that interpretation lengthened appointment times and varied in accuracy, thoroughness, and ability to accommodate diverse accents and hearing abilities, prompting a preference for family members as ad hoc interpreters (capability, opportunity):

“One appointment, originally half an hour, became an hour, and it’s not possible to be very detailed. …We translate ourselves and communication with the doctor becomes easier … we find [interpretation] really a waste of time, not very accurate. What I want to say isn’t necessarily what is actually said.” (Cantonese-speaking caregiver)

“I will say I don’t use the interpreter team much … because my dad doesn’t hear well and sometimes we have people from other countries … and there is an accent. I mean, it’s difficult for my dad, you understand?… It becomes much easier and faster for me to do it myself.” (Spanish-speaking caregiver)

## Discussion

In this qualitative study of interviews with Cantonese- and Spanish-speaking patients with serious illnesses or their caregivers, we identified 3 themes regarding facilitators and barriers before, during, and between telehealth visits. Participants perceived telehealth visits as the recommended default and had unvoiced questions regarding other visit types. Between-visit communication via the electronic patient portal was limited by a lack of non-English language translation. Finally, language concordance with clinicians and longitudinal relationships with interpreters facilitated telehealth visits by increasing understanding, comfort, and alleviating fears and perceived privacy concerns.

Our study identified a preference for language-concordant clinicians. Research has shown that language concordance is associated with higher-quality patient-clinician communication in both in-person primary and urgent care^11,12,19,20,21,22^ and may facilitate better communication during telehealth visits.^6^ In a study evaluating oncology visits, there were more partnership- or facilitation-related comments in the group of patients with PLOE seen by language-concordant clinicians compared with the group that used certified interpreters.^23^ Thus, language concordance may facilitate rapport building––an essential component of the patient-clinician relationship in serious illness care—and overcome perceived emotional distance imposed by telehealth. Unfortunately, there is a mismatch between the language composition of palliative care clinicians and patients.^24^ These findings suggest that efforts to increase linguistic diversity in the health care workforce are vital.

We identified issues unique to interpretation during video visits. We found that developing longitudinal relationships with interpreters could facilitate comfort in discussing difficult topics and allay privacy concerns. However, in our practice, video visits typically use randomly assigned external interpreters rather than consistent staff interpreters. These logistical challenges experienced in our practice, which was an early adopter of telehealth even before the COVID-19 pandemic, are likely to affect other outpatient practices too. The findings of our study suggest that integrating certified interpreters into our clinical teams and pairing patients with small teams of interpreters would facilitate building longitudinal relationships. We also learned that telehealth interpretation can be particularly challenging for people with communication barriers, such as being hard of hearing or hypophonic (challenges that are more common in patients with serious illnesses), requiring family members to act as primary communicators and interpreters. Family members may be uniquely positioned to serve as cultural brokers and patient advocates,^25^ facilitating increased patient comfort and autonomy^26,27,28^; however, they also may purposefully or inadvertently omit content to shelter patients from bad news and may experience undue burden in order to simultaneously process information as a caregiver and convey it to a loved one.^15,29^ Furthermore, studies have demonstrated that using ad hoc interpreters instead of certified interpreters was associated with worse outcomes.^15,16^ Therefore, interventions that increase the efficacy of interpreter use (eg, supplementing video with other digital health modalities such as text to speech or closed captioning) need to be explored.

Our findings on interpretation inaccuracy align with prior studies showing frequent, clinically significant errors in the interpretation of serious illness communication.^30,31^ Mechanistically, interpreters have identified a lack of verbatim translation for serious illness–related terms (eg, *palliative care*) as a challenge.^32^ Efforts to promote linguistic equity as part of providing serious illness care to people with PLOE^33^ include interpreter training programs in palliative care concepts^34^ and work by community-based organizations to translate palliative care terms.^35^ However, broader implementation across languages and standardized use of culturally appropriate translated terms are needed. In parallel, premeetings between clinicians and interpreters, including explicit discussions regarding the content of the visit, have been shown to improve the quality of communication.^10,36^ Extending visit time for people with PLOE, both for prebriefs and debriefs and for the extra time required for interpretation, may be helpful and facilitated by time-based billing. Finally, compared with consecutive interpretation, which occurs after the speaker has completed speaking, simultaneous interpretation, where interpretation is provided within milliseconds of the original speech, has been associated with higher patient satisfaction.^37^ In parallel, large-language models (LLMs) were shown to accurately translate written discharge instructions.^38,39^ Taken together, further work that explores different modes of interpretation and incorporation of LLMs to facilitate real-time interpretation will be a valuable future direction.

In this study, patients with PLOE expressed difficulty accessing their telehealth visits and the electronic patient portal, necessitating caregiver assistance. We also learned that participants defaulted to the visit type scheduled by clinic staff without expressing their preferences, possibly secondary to barriers to communication through the patient portal, where upcoming appointment information is shared. Previous studies also identified lower digital literacy^40,41^ and limited device and data access as barriers to video visits for people with PLOE.^6^ Although caregivers typically assist people with PLOE with health care navigation,^42^ caregiver access to patient portals is low.^43,44^ As health care processes become increasingly digital, ensuring accessibility of such processes for people with PLOE and their caregivers through language-inclusive digital health tools is crucial.

Our findings highlight key, actionable steps that clinicians, clinics, and health systems can take to improve equity and quality of care for this population. These steps include extending outpatient visit times to incorporate prebrief and debrief time with interpreters, incorporating a team of consistent interpreters to work with clinicians caring for people with serious illness, and increasing the reach of existing training for interpreters in key serious illness care concepts. On a broader scale, our findings suggest that integrating interpreters into interdisciplinary teams across the health care system would foster a more cohesive, language-equitable team that can build longitudinal relationships with patients. The results of this study also suggest that linguistically diversifying the health care workforce would address barriers fundamentally. Health systems also should prioritize language equity in patient portals and explore how artificial intelligence might facilitate patient-clinician messaging in all languages. Finally, developing and standardizing language for serious illness and palliative care concepts could improve the clarity of communication with people with PLOE.

### Limitations

This study has limitations. It was conducted in 2 clinics within a quaternary health system with a relatively high telehealth uptake and may not be generalizable to other patient populations. We focused on Cantonese- and Spanish-speaking participants because they are the 2 largest language groups in the US aside from English speakers. Although we identified similar themes across these 2 groups, there were notable differences in the mean age of the participants (Cantonese-speaking participants were older) and the rate of electronic patient portal use (Cantonese-speaking patients had lower rates). Because patients are referred to our clinic, differences in patient characteristics (eg, age and race and ethnicity) could reflect various referral process factors and race- and ethnicity-related disease factors beyond this study’s scope. The older age of Cantonese-speaking patients may directly contribute to their lower use of digital tools, although this relationship requires further investigation. Overall, future work that explores each language group more deeply, as well as other language groups, may uncover further opportunities to provide equitable serious illness care across the US.

## Conclusions

In this qualitative study of 20 semistructured interviews, Cantonese- and Spanish-speaking participants experienced barriers to serious illness care via telehealth before, during, and between visits. These findings underscore the need for interventions to promote equitable telehealth care for people with serious illnesses and PLOE, such as training more language-concordant clinicians, incorporating interpreters into interdisciplinary teams, and ensuring access to patient portals in languages other than English.

## Notes

Supplement 1.
eMethods

Supplement 2.
Data Sharing Statement
