# Supplementary material for: Telehealth Care for People With Serious Illnesses and Preferred Languages Other Than English
Source: JAMA Netw Open. 2025 Sep 3;8(9):e2529880. doi: 10.1001/jamanetworkopen.2025.29880 (PMC12409582; doi:10.1001/jamanetworkopen.2025.29880)
Supplement: Supplement 1. — eMethods [file jamanetwopen-e2529880-s001.pdf]

Supplemental Online Content

Wang Y, Sudore RL, Zapata C, et al. Telehealth care for people with serious illnesses and preferred languages other than English. *JAMA Netw Open*. 2025;8(9):e2529880. doi:10.1001/jamanetworkopen.2025.29880

eMethods

This supplemental material has been provided by the authors to give readers additional information about their work.

© 2025 Wang Y et al. *JAMA Network Open*.

eMethods

Participant selection
Participants were selected through consecutive sampling of patients via telephone outreach conducted by language-concordant study staff. 59 people that we tried reaching out to did not participate. Of those 59: 33 were people we were never able to reach by phone, 16 were interested but things came up that prevented us from actually doing the interview (e.g., schedules didn't line up between interviewer and participant, participant had to cancel last minute etc), and the remaining 10 said they could not because they were too busy.

Research team and reflexivity
The semi-structured interview guides were pilot tested with members of our Community Advisory Board. All interviews were 30-45 minutes in duration, conducted via telephone by JG, MQ, CH, and CPV. Each Spanish interview transcript was coded by language-concordant coders (JG, CPV, SN, and CZ) and a combination of culturally concordant (JG, CPV) and non-concordant (SN, CZ) coders. The Cantonese transcripts were translated into English by professional translator KH (see eMethod section *Data collection*), reviewed by language and culturally concordant research staff CH, and independently coded by language-discordant research staff (SN, VL, MQ).

At the time of the study:

- YW: Chinese, culturally concordant, Mandarin-speaking, woman, internal medicine doctor, and researcher
- RLS: White, culturally and language-discordant, woman, palliative care doctor, geriatrician, and researcher
- CZ: White, culturally discordant, Spanish-speaking woman, palliative care doctor
- RM: Latino/Hispanic, culturally concordant, language-discordant medical student at UCSF
- JG: Latino/Hispanic, culturally concordant, Spanish-speaking, woman, medical assistant
- MQ: White, culturally and language-discordant, woman, clinical research coordinator
- CH: Chinese, culturally concordant, Cantonese-speaking woman, hospice liaison and research assistant
- CPV: Latino/Hispanic, culturally concordant, Spanish-speaking, woman, activities director at community-based organization
- SZP: White, culturally and language-discordant, man, palliative care doctor
- CRL: White, culturally and language-discordant, woman, researcher
- CSR: White, culturally and language-discordant, woman, palliative care doctor and researcher
- SN: Middle Eastern, culturally discordant, Spanish-speaking woman, palliative care doctor and researcher

Data collection
The interviews were recorded using either Zoom or a native voice recording app on Windows or iOS computers and transcribed for analysis. Interviews in Cantonese were translated into English before analysis by KH. KH is a certified bilingual newspaper reporter and editor for one of the largest local Chinese newspapers in San Francisco and has 13 years of experience translating government documents, press releases, and legal terminology for publication. The translated material was also reviewed by CH, a bilingual research staff member and interviewer for Cantonese participants. Interviews in Spanish were analyzed in Spanish. Interviewers were recruited by SN through prior research collaborations. All interviewers had previously been trained in and conducted semi-structured interviews. At the start of the study, SN provided refresher training to all interviewers, then listened to their first interview and provided feedback. Interviewers did not have any relationship with participants prior to study commencement.

Qualitative Analysis
We (SN, CZ, RM, JG, VL, MQ, CH, CPV) thematically coded a subset of interviews (2 per language group) as a group to create the Spanish and Cantonese interview code books. We generated codes both deductively (based on COM-B domains) and inductively through line-by-line analysis and discussion. We

© 2025 Wang Y et al. *JAMA Network Open*.

observed that 94% of the codes were the same between the Spanish and Cantonese codebooks. Twenty-five percent of interviews were subsequently double-coded by CZ, RM, JG, VL, MQ, with very few additional codes arising and consequent edits to the code books. Given the persistently significant amount of overlap, we combined the codebooks into a single codebook. All interviews were then triple-coded by SN.

© 2025 Wang Y et al. *JAMA Network Open*.
