# Supplementary material for: Telehealth Care for People With Serious Illnesses and Preferred Languages Other Than English
Source: JAMA Netw Open. 2025 Sep 3;8(9):e2529880. doi: 10.1001/jamanetworkopen.2025.29880 (PMC12409582; doi:10.1001/jamanetworkopen.2025.29880)
Supplement: Supplement 2. — Data Sharing Statement [file jamanetwopen-e2529880-s002.pdf]

Data Sharing Statement

Wang. Telehealth Care for People With Serious Illnesses and Preferred Languages Other Than English. *JAMA Netw Open*. Published September 03, 2025.
doi:10.1001/jamanetworkopen.2025.29880

Data
Data available: This patient-related information is not available for sharing. Upon request, a codebook can be made available to replicate analyses.
